# Mt. Sinai Hospital Reports

**Published:** 1902-04

**Authors:** 


Mt. Sinai Hospital Reports. Volume II. For 1899 and 1900. Edited by Paul F. Mundé, M. D. New York. 1901.

In 1899 was published Volume I. of the Mt. Sinai Hospital Reports and the number of cases commented on. Now comes Volume II., covering the work done for the years 1899 and 1900, edited in the same careful painstaking way, so characteristic of everything done by its editor, Dr. Paul F. Mundé, of New York. The statistics giving the number of patients treated during the year, the number discharged cured, improved or that died, shows that better work was accomplished in 1900 than in 1899, and yet more patients were received in 1898.

The report embraces the following papers and contributions: A case of acromegaly; diabetes mellitus with excessive polyuria; hyperidrosis, by J. Rudisch; A case of diabetes insipidus cured by suggestion, by J. Rudisch; A case of typhoid fever, complicated by a broncho-pneumonia, simulating typhus fever, by J. Rudisch; Statistics of cases of diabetes in the Mount Sinai Hospital during the years 1890-1900, by Julius Rudisch and Edward A. Aronson; On the relationship of fistula in ano to pulmonary tuberculosis, by Alfred Meyer; Typhoid fever and pharyngeal diphtheria, by Morris Manges; Acute lobar pneumonia, followed by purulent pericarditis; pericardotomy, recovery, by Morris Manges, and Howard Lilienthal; A case of exophthalmic goitre with diabetes mellitus and pigmentation, by Morris Manges; A case of rheumatoid arthritis associated with exophthalmic goitre in a girl sixteen years old, by Morris Manges; Pneumonia and the acute hemorrhagic diathesis, by N. E. Brill; Systemic pyocyaneus infection. A critical view of the recorded cases, with the report of a case secondary to a staphylococcemia, by N. E. Brill and E. Libman; A contribution to the subjects of chronic interstitial nephritis and arteritis in the young, and family nephritis, with a note on calcification in the liver, by N. E. Brill and E. Libman; Gliosarcoma of the base of the brain pressing upon and in front of the left lobe of the cerebellum, with remarks on the diagnosis of cerebellar disease, by Henry W. Berg; Purpura hemorrhagica, by D. H. Davison; Statistics of children's diseases, by B. Scharlau; Report of the department of general surgery for 1899, by Arpad G. Gerster, A. A. Berg, A. V. Moschcowitz and Joseph Wiener, Jr.; Report of the department of general surgery for 1900, by Arpad G. Gerster, A. A. Berg and A. V. Moschcowitz; Report of the second surgical division, by Howard Lilienthal, Joseph Wiener, Jr., and Charles A. Elsberg; Simultaneous fracture of the vault and base of the skull—operation and recovery, by Howard Lilienthal; Gynecological service, by Paul F. Mundé, and Joseph Brettauer; Three cases of primary carcinoma of the vulva, by Joseph Brettauer; On tubercle of the choroid in conjunction with tubercular meningitis, by E. Gruening; The early recognition of choroidal sarcoma, by E. Gruening; Two cases of otitic brain abscess, operation, recovery, by E. Gruening; A series of mastoid operations, Charles H. May; Statistics of eye and ear service, by Carl Koller; A case of thrombophlebitis of the left sigmoid sinus masking a latent brain abscess in the left temporo-sphenoidal lobe, both arising from chronic otitis media, by Carl Koller; Report of anesthesias, 1900, by Eugene H. Eising; A case of dermoid cyst of the mediastinum, with remarks upon the etiology and embryology, and a survey of recent cases, by F. S. Mandlebaum.

The staff of Mount Sinai are to be congratulated on their work and on the able manner in which it is laid before the profession.

